# Rapid response

**DOI:** 10.7554/eLife.57105

**Published:** 2020-04-17

**Authors:** Jon Hughes

**Affiliations:** Department of Plant Physiology, Justus Liebig UniversityGiessenGermany

**Keywords:** phytochromes, SFX, Deinococcus radiodurans, initial photoresponse, free-electron laser, Other

## Abstract

Extremely short X-ray pulses from a free-electron laser are helping to clarify how phytochromes respond to light, but puzzles remain.

**Related research article** Claesson E, Wahlgren WY, Takala H, Pandey S, Castillon L, Kuznetsova V, Henry L, Panman M, Carrillo M, Kübel J, Nanekar R, Isaksson L, Nimmrich A, Cellini A, Morozov D, Maj M, Kurttila M, Bosman R, Nango E, Tanaka R, Tanaka T, Fangjia L, Iwata S, Owada S, Moffat K, Groenhof G, Stojković EA, Ihalainen JA, Schmidt M, Westenhoff S. 2020. The primary structural photoresponse of phytochrome proteins captured by a femtosecond X-ray laser. *eLife* **9**:e53514. doi: 10.7554/eLife.53514

It might surprise you, but plants don't just use light for photosynthesis – like we do, they also use it to collect information about their environment. For this they use a photoreceptor protein called phytochrome that has a number of remarkable properties. For example, it regulates almost a quarter of the plant genome, giving it control over many aspects of plant development. Little wonder, then, that researchers are keen to understand its workings. Now, in eLife, a team of researchers from Sweden, Finland, the USA and Japan report the results of experiments in which they have used ultrashort pulses of X-rays from an expensive new tool, a free-electron laser, to record how the structure of the molecule changes after it absorbs a photon of light (Claesson et al., 2020).

The usual way to determine the 3D structure of a protein is to crystalize it, then measure how these crystals diffract X-rays and, finally, calculate the structure of the protein from the diffraction data. A free-electron laser produces incredibly powerful X-rays in extremely short flashes, allowing it to follow how the structure of a protein changes with time. In experiments conducted at the SACLA facility in Japan – an instrument nearly a kilometre in length – Claesson et al. used ultrashort pulses of red light from a titanium-sapphire laser to excite phytochrome protein crystals, and similarly short pulses of X-rays from the free-electron laser to determine the protein structure one picosecond (10^−12^ seconds) and 10 picoseconds after excitation. Not bad.

Phytochrome is remarkable because it doesn't just send a one-off signal when it absorbs a photon of light, it remains switched on and continues to signal for hours. That helps to make phytochrome an exceedingly sensitive light detector, but there is more to it than that. Phytochrome is most sensitive to red light, but when it absorbs a photon of red light, it changes colour and becomes sensitive to far-red light, a region of the spectrum that the human eye can hardly see. Remarkably, through this shift, phytochrome allows the plant to perceive the leaves of nearby competitors and change its growth strategy accordingly.

We have known for nearly 60 years that phytochrome contains a deep blue pigment molecule called a bilin to absorb light. Bilins comprise a row of four pyrrole rings (see Figure 1) and can absorb light very efficiently. In the early 1980s Wolfgang Rüdiger and co-workers in Munich suggested that when the bilin absorbs a photon, the final pyrrole ring (the *D*-ring) rotates, somehow using the energy to 'kick' the phytochrome into action (Rüdiger et al., 1983). Indeed, several amino acids near the *D*-ring flip over too (Essen et al., 2008; Yang et al., 2008). Then, six years ago, Sebastian Westenhoff of the University of Gothenburg, Janne Ihalainen of the University of Jyvaskyla and co-workers electrified the phytochrome field by showing that a whole section of the protein known as the 'tongue' refolds completely upon light activation: this involves a sheet-like structure in the phytochrome being torn apart and reforming as a helix, perhaps setting the signalling machinery of the cell in motion (Takala et al., 2014).

**Figure 1.**
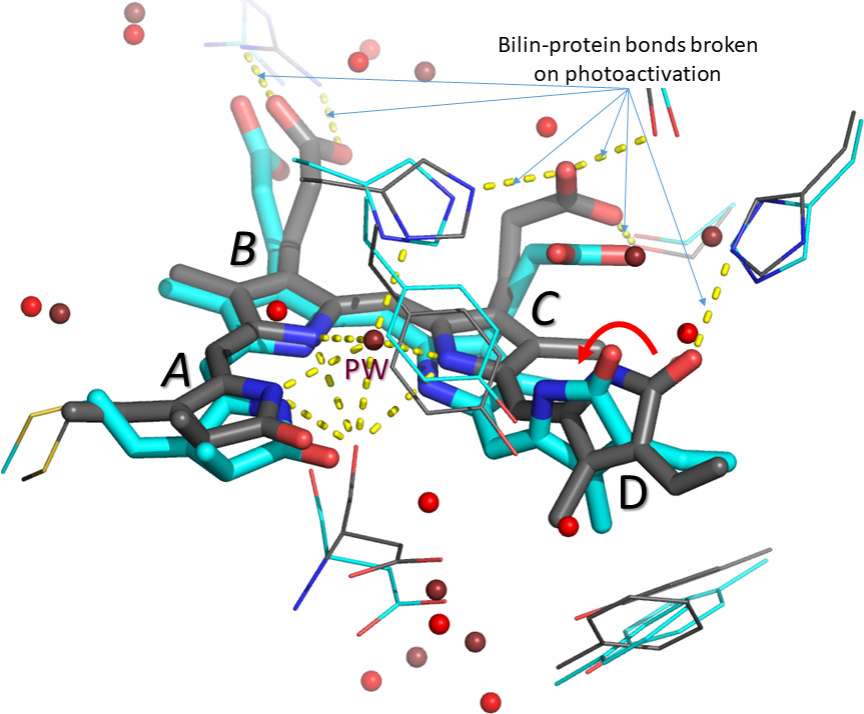
The bilin inside the phytochrome before and after photoactivation. The four rings of the bilin are labelled *A-D.* Carbon atoms before and one picosecond after photoactivation are shown in grey and cyan, respectively. Water molecules before and after photoactivation are shown in deep red and bright red, respectively. Otherwise, oxygen, nitrogen and sulphur atoms are shown in red, blue and yellow, respectively. The ~50° rotation of the *D*-ring is indicated by the red arrow. The pyrrole water molecule (PW) above the nitrogen atoms of the *A-, B-* and *C*-rings disappears on photoactivation. Hydrogen bonds (yellow dashes) between the bilin and amino acid side chains in the rest of the phytochrome are also broken. Figure prepared by the author using PyMol from data provided by Claesson et al.

In the latest work Westenhoff and Ihalainen – in collaboration with Marius Schmidt (University of Wisconsin-Milwaukee) and Keith Moffat (University of Chicago), and with Elin Claesson, Weixiao Yuan Wahlgren and Heikki Takala as joint first authors – report that, just as expected, the action starts at the *D*-ring (Claesson et al., 2020). But it's not quite as straightforward as that, because they show that the movement begins within a picosecond of the photon being adsorbed, whereas Karsten Heyne and co-workers in Berlin showed that at least in some phytochromes the movement happens a good deal later, even after 30 picoseconds (Yang et al., 2012). This is an interesting paradox that needs explaining. Unfortunately, the structure obtained for 10 picoseconds after adsorption is not as clear as that obtained after one picosecond, so it cannot shed light on the discrepancy between the latest work and the results of Heyne and co-workers.

There are a number of other surprises and puzzles. First, the one picosecond structure implies that the *D*-ring has rotated anti-clockwise by about 50°, whereas in its final position after photoactivation the ring is rotated by almost 180°; we also expected the rotation to be clockwise in this type of phytochrome. Perhaps what is being seen is just the first phase of a longer process. Second, a water molecule that is positioned exactly above the nitrogen atoms in the *A-*, *B-* and *C-*rings before photoactivation is missing from the one picosecond structure – and it's not clear where it has gone. Third, the *A*- and *C*-rings have moved downwards. Both the *B*- and *C*-rings have acidic side chains that associate with nearby amino acid side chains in the 'dark state' before photoactivation. It is no surprise that these connections are broken during photoactivation, but according to the new data, this too happens within a picosecond. I don't think anyone was expecting so much to happen this quickly – and it needs explaining.

The unexpected nature of some of the new results means that it will be necessary to rule out some possible technical problems. For example, the red laser flash is so bright that the bilin might have absorbed not one but two photons: that would lead to very strange effects, totally unrelated to what happens in normal daylight. Moreover, Claesson et al. studied only a small fragment of the complete phytochrome molecule: it is not clear to what extent the fragment behaves like the real thing. It is also ironic that this fragment is missing the tongue region that Westenhoff and Ihalainen proposed in 2014 to be the central player in signalling.

The new paper is clearly not the last word on the photoactivation of phytochrome, but Ihalainen, Westenhoff and co-workers have – for the second time in six years – presented us with a feast of unexpected information and novel ideas, setting the scene for further studies and, probably, heated discussions. This is the way science progresses.
